# Individually distinctive features facilitate numerical discrimination of sets of objects in domestic chicks

**DOI:** 10.1038/s41598-020-73431-3

**Published:** 2020-10-02

**Authors:** Rosa Rugani, Maria Loconsole, Francesca Simion, Lucia Regolin

**Affiliations:** 1 Department of General Psychology, University of Padua, via Venezia 8, 35100 Padua, PD Italy; 2 Department of Psychology, University of Pennsylvania, Philadelphia, PA USA; 3 Department of Developmental Psychology and Socialization, University of Padua, Padua, Italy

**Keywords:** Psychology, Zoology

## Abstract

Day-old domestic chicks approach the larger of two groups of identical objects, but in a 3 vs 4 comparison, their performance is random. Here we investigated whether adding individually distinctive features to each object would facilitate such discrimination. Chicks reared with 7 objects were presented with the operation 1 + 1 + 1 vs 1 + 1 + 1 + 1. When objects were all identical, chicks performed randomly, as expected (Experiment 1). In the remaining experiments, objects differed from one another due to additional features. Chicks succeeded when those features were differently oriented segments (Experiment 2) but failed when the features were arranged to depict individually different face-like displays (Experiment 3). Discrimination was restored if the face-like stimuli were presented upside-down, disrupting global processing (Experiment 4). Our results support the claim that numerical discrimination in 3 vs 4 comparison benefits from the presence of distinctive features that enhance object individuation due to individual processing. Interestingly, when the distinctive features are arranged into upright face-like displays, the process is susceptible to global over local interference due to configural processing. This study was aimed at assessing whether individual object processing affects numerical discrimination. We hypothesise that in humans similar strategies aimed at improving performance at the non-symbolic level may have positive effects on symbolic mathematical abilities.

## Introduction

Humans perceive the world as a multitude of discrete objects rather than as a continuous flow of visual information. Objects come in all sizes, shapes and kinds. Some are especially salient, such as people or animals; others are minute and apparently negligible, such as pebbles or leaves. Individuals’ daily interactions with the environment are determined by factors such as the way they perceive, categorise, identify, localise and track objects. Often, they also quantify objects and may enumerate them. However, object enumeration is not an independent process; in fact, it is intimately intertwined with object perception and identification. This study addresses how these processes, particularly object identification and enumeration, interact.

All objects are characterised by unique and coherent spatiotemporal features^1,2^. For example, at any given moment, each object occupies a distinctive space and position which cannot be penetrated by other objects. The same object cannot simultaneously reside in more than one location. Objects are also characterised by intrinsic properties (i.e. their key features) and category belonging^1^, and both are usually expected to be preserved over time. Individual processing of some objects (i.e. their individuation) could be based on one or more of the characteristics mentioned above and would facilitate tracing the object’s identity through space and time. Object individuation occurs for relevant objects, such as social agents, for which individual-level categorisation occurs spontaneously. Most objects, however, are only processed at the category level. For instance, a single leaf or rock is just one of the many leaves or rocks people experience unless they are botanists or geologists^3^. Perceptually salient specimens (e.g. a rock of a peculiar colour or shape) can still trigger individual processing even in non-experts, and, most likely, also in non-humans^4^. The Makapansgat pebble is thought to have received special attention from *Australopithecus africanus* individuals 3 million years ago, probably because of its natural chipping, which resembles a human face^5^. Only speculations can be made concerning the origin of face-processing abilities in our pre-human ancestors. However, modern humans are experts in recognising faces at an individual level by processing not only the characteristics of the facial features but also their configural properties. These comprise first-order relations (i.e. the triangular disposition of the inner elements—two eyes and a mouth) and second-order relations (i.e. the spacing among them)^6^. From birth, babies detect and recognise individual faces on the basis of global configuration and inner features^7–10^. However, humans are not the only species sensitive to schematic faces^11,12^ or illusory facial structures in inanimate objects^4^. Objects bearing salient features are probably processed individually and remembered, located and tracked more efficiently. Object individuation, moreover, constitutes the initial process that allows individuals to determine the number of objects and establish their numerical identity^13^.

The present study is aimed at assessing whether individual object processing affects numerical discrimination. We tested newly hatched domestic chicks, a model species that allows for full control of previous experiences, through the manipulation of post-hatching exposure to the experimental stimuli.

Numerical competence in non-human species is widespread and well attested. Several species can master a variety of number processing skills^14^, which are built on non-verbal number systems^15^ and considered the roots of non-symbolic numerical comprehension in all animals. One such ancestral mechanisms, the Object File System (OFS) identifies and tracks objects by representing each object as a separate file in the working memory^16^. The overall number of object files that can be simultaneously attended to and held in the working memory is limited (usually ≤ 3 per group). The OFS also allows individuals to estimate numerousness by evaluating the number of files stored in the working memory^16^. Computation of larger (≥ 4) sets is usually supported not by this system, but by the Analogue Magnitude System (AMS), a dedicated number system which operates in a ratio-dependent manner unaffected by set size limits^17^.

Day-old domestic chicks reared with identical objects, when presented with sets of 2 vs 3, 1 vs 4, 1 vs 5 and 2 vs 4 objects that disappear one-by-one behind separate panels spontaneously inspect the panel occluding the larger set^18,19^. Chicks discriminate between large numerousnesses, such as 6 vs 9 and 5 vs 10^20^ but fail with 3 vs 4^21^. This is consistent with findings on infants that report an upper limit of 3 in tracking and representing multiple occluded objects^22–24^. Failure could occur because the ratio is difficult (too small) to discern using the AMS or because the number of elements exceeds the OFS limit. Interestingly, the use of cognitive strategies, such as grouping, improves numerical discrimination (probably by increasing object storage in the working memory) in infants^25–27^ and chicks^21,28^. Grouping strategies in particular may be crucial in the absence of linguistic labels or when stimuli only allow for category-level processing, such as when objects are identical and cannot be distinguished at the individual level.

**Figure 1:**
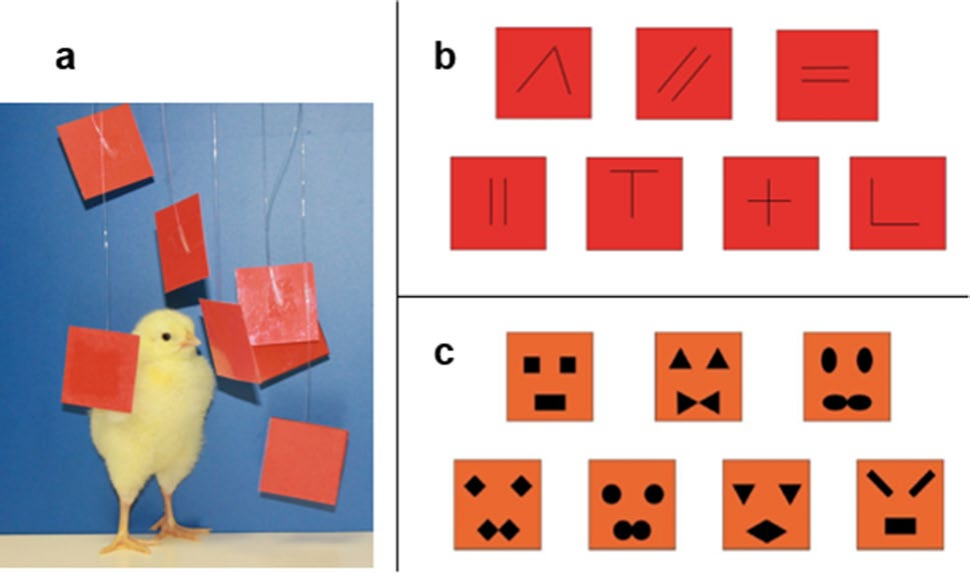
(**a**) A chick with all identical square stimuli used in Exp. 1 (for illustrative purposes, stimuli are closer together than in the actual experimental condition); (**b**) stimuli used in Exp. 2; (**c**) stimuli used in Exp. 3 (the same stimuli were presented upside-down, in Exp. 4).

In this study we manipulated array heterogeneity to assess discrimination of 3 vs 4 objects. We reared newly hatched domestic chicks with 7 objects for 3 days. On Day 4, the chicks underwent a 1 + 1 + 1 vs 1 + 1 + 1 + 1 (3 vs 4) discrimination. Each of the 7 objects was shown to the chick and then made to disappear: 3 objects behind one panel and 4 objects behind another. In Experiment 1, we used all identical objects (red squares, Fig. 1a), and we expected the chicks to be unable to discriminate between them, consistent with previous evidence^21^. In Experiment 2, each square differed from the others in terms of the relative orientation of two black segments (Fig. 1b); such features are known to support individual processing and object individuation in domestic chicks^29,30^. We hypothesised that adding individually distinctive features would result in improved performance in the 3 vs 4 discrimination^31,32^. Experiment 3 employed stimuli designed to be especially salient for the young chicks: face-like configurations composed of three inner features arranged triangularly (Fig. 1c). Newly hatched chicks preferentially attend to artificial stimuli representing schematic faces rather than non-face-like stimuli^11^. Therefore, we expected these stimuli to attract the chicks’ attention. Moreover, each configuration was unique: it differed from all others in terms of shape, size and relative distance of the three features.

In this species, exposure to natural or artificial objects during the first days of life triggers perceptual learning through filial imprinting. This is an early mechanism dedicated to establishing the rapid and effective processing of individuals in precocial bird species^33^. Imprinted chicks recognise familiar objects and display social attachment towards them. Chicks react to separation with strong social reinstatement responses, such as searching, and they follow and rejoin the larger subset of their naturalistic or artificial “companions”^18^ . Moreover, individual recognition among chicks relies on conspecifics’ facial features^8,34^. Therefore, we expected face-like stimuli to trigger selective attention and individual processing, facilitating chicks’ numerical performance in spite of the higher level of complexity of those stimuli (presence of three detached features).

In Experiment 4, we used the same stimuli as in Experiment 3 but presented them upside-down to disrupt configural processing and to disentangle between the chicks’ use of local and global cues. Based on previous literature^11^, we expected the chicks to be sensitive to this manipulation in a manner similar to the well-known inversion effect described in human observers.

## Results

**Figure 2:**
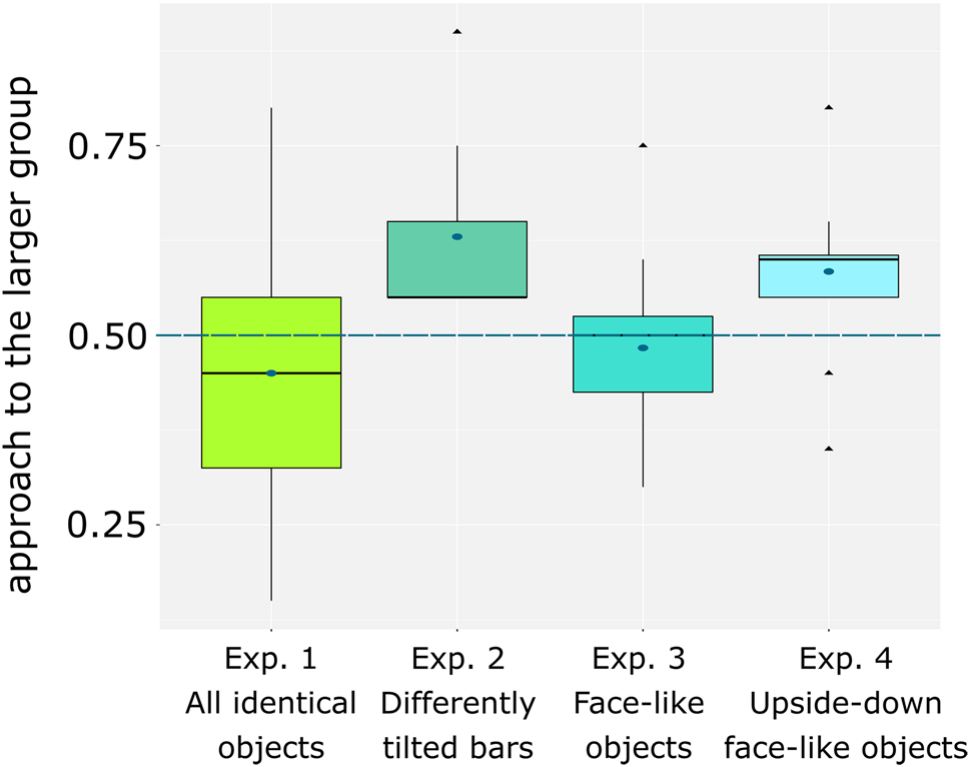
Probability of the chicks’ approaching the larger set in each of the four experiments. The dashed line indicates the chance level (y = 0.50). For each experiment, the black bar represents the median; the central dot represents the mean.

We conducted statistical analyses in R 3.5.3^35^. For each test trial, we scored the first panel circumnavigated by the chick. The number of trials in which the chicks circumnavigated the panel hiding 4 objects (regarded as the correct response) was calculated. As the data were not normally distributed (Shapiro–Wilk normality test, p < 0.05), we used a one-sample Wilcoxon test. The results of all experiments are reported in Fig. 2.

In Experiment 1, the chicks did not preferentially circumnavigate any panel: n = 15; M = 0.450; ES = 0.050; V = 33, p = 0.400, r = − 0.226.

The chicks’ performance did not differ from chance when considering only the first five trials: M = 0.387; ES = 0.066; V = 37, p = 0.195, r = − 0.341. Hence, their overall performance did not seem to depend on a progressive decrease in motivation or attention, which might have occurred during testing^21^. The chicks seemed unable to discriminate between 3 and 4 identical objects when these were presented and hidden in spatiotemporal discontinuity.

In Experiment 2, the chicks preferred the panel hiding the larger set: n = 15; M = 0.63, ES = 0.032; V = 120, p value < 0.001, r = 0.896. The chicks’ performance was already significant when considering only the first five trials, ruling out the possible role of progressive learning, which might have occurred during training (M = 0.61; ES = 0.032; V = 107, p value = 0.005, r = 0.733). Lastly, there was no difference between the performance in the first five trials and the last five trials (two-sample Wilcoxon test, W = 129, p = 0.496, r = − 0.257). The use of perceptually heterogeneous stimuli during rearing and testing could significantly improve the chicks’ mathematical performance.

In Experiment 3, the chicks did not circumnavigate the panel hiding the larger set of objects when these were characterised by three features positioned in a triangular, face-like, fashion: n = 15; M = 0.483; ES = 0.028; V = 27, p = 0.6233, r = − 0.138. The chicks’ performance did not differ from chance in the first five trials: M = 0.467; ES = 0.050; V = 41, p value = 0.2638, r = − 0.297. This suggests that the presence of three rather than two features or their spatial arrangement may have negatively affected object individuation and, consequently, the chicks’ performance.

In Experiment 4, chicks successfully discriminated in the 3 vs 4 comparison when the stimuli used in Experiment 3 were turned upside down to disrupt configural processing: n = 15; M = 0.584; ES = 0.025; V = 106, p = 0.009, r = 0.682. The effect was already present even when restricting the analysis to the first five trials (M = 0.653; ES = 0.041; V = 106.5, p = 0.007, r = 0.702). There was no difference between the performance in the first five trials and in the last five trials (W = 127, p = 0.557, r = 0.272).

## Discussion

We hypothesised that the chicks’ performance in the 3 vs 4 discrimination would benefit from the presence of features allowing for object identification (i.e. recognition at the individual level). In the absence of such features (i.e. when all objects were identical), the chicks failed the 3 vs 4 discrimination (Experiment 1), which is consistent with previous results^21^. Experience with a set of heterogeneous objects in which each stimulus presents individually distinctive features might reduce the cognitive load involved in creating representations of the stimulus sets, facilitating the comparison between the groups. In Experiment 2, in which each object was characterised by a unique arrangement of black segments depicted on both sides of each stimulus, the chicks discriminated between 3 and 4. Such uniquely distinctive features are proved sufficient to support individual discrimination in chicks^29,30^. Here, the presence of distinctive features may have enhanced object individuation, making objects individually recoverable even when grouped together and facilitating increased information storage in memory^36–39^. As a result, each object could be identified, tracked and remembered more effectively.

The stimuli employed in Experiment 3 depicted different face-like displays on both sides of each square. We expected these stimuli to be especially salient for young chicks and to be rather effective at triggering individual processing and, thus, supporting numerical discrimination. In fact, individual recognition of conspecifics in chicks specifically depends on face and head features^40^; moreover, newly hatched chicks preferentially attend to artificial stimuli that resemble face schematics than to non-face-like stimuli^11^. Therefore, Experiment 3’s stimuli should support numerical discrimination in spite of their higher complexity (there were three detached black features rather than one or two features, as in the stimuli used for Experiment 2). The stimuli for Experiment 3 provided local (i.e. the three inner features, which differed for each “face”) and configural (i.e. the triangular, face-like arrangement of the inner features) levels of analysis.

Contrary to our expectations, however, the chicks failed to discriminate between 3 and 4 in Experiment 3. Several hypotheses might account for this unexpected outcome:

1. The stimuli were too complex for the chicks to process at the individual level. Chicks may be unable to process and identify objects with three detached features. This is unlikely, however, as the discrimination of each object could still be based on a single feature such as the “mouth”, or one “eye” without the need to refer to first- or second-order information. Moreover, chicks are usually quite capable of detecting fine perceptual differences in visual stimuli, as is also clear from the results of Experiment 2.
2. The face-like stimuli were only processed at the category detection level of first-order relations. Global processing might have preceded and dominated local processing. As a result, the chicks might have neglected the differences present in the stimuli at the local, individual level. Recent evidence shows that chicks trained in a discrimination learning task prefer local over global information processing^41^. However, our chicks did not undergo any formal training, and chicks exposed early in life to complex configurations similar to the conditions of the present study are known to prioritise global over local processing^42^.
3. Our face-like displays may have provided ambiguous information. They may have been detected as just face-like enough to be processed at the configural level of first-order relations (two eyes above the mouth^6^), but they may have lacked the specific critical properties needed to support intra-category or individual level processing. For example, in chicks, face-like stimuli with square outlines are less effective than those with round outlines for the discrimination of face-like displays^11^. In addition, our stimuli had two features at the top and one at the bottom, but the overall surface at the top and at bottom areas was controlled (i.e. the “mouth” was always the sum of the surface of both “eyes”). Hence, the vertically (up-down) asymmetrical distribution of the inner features typical of a face, which is critical for obtaining facial preference in human newborns^9^, was present regarding the number of the features, but not regarding the surface area amount. When designing the study, this did not seem to constitute a possible issue, as chicks, unlike human babies, respond to up down balanced face-like configurations and prefer a face-like display to a non-face-like display^11^. Nevertheless, previous studies on chicks only tested for their spontaneous preference between face-like and non-face like displays. Individual processing of different face-like configurations has never been tested in chicks. Consequently, this process might require the presence of a quantitative “top-heaviness”, as well as an asymmetrical distribution in the number of inner features (i.e. two elements at the top and one element at the bottom).

Although the last hypothesis can only be investigated by designing new stimuli and new studies, the first two can be tested experimentally to disentangle between the two alternatives. We did this in Experiment 4 by testing a new group of chicks using the stimuli from Experiment 3, but turned upside-down. Chicks discriminate between upright and upside-down faces^11^; for evidence regarding the inversion effect with a point-light biological motion display, see^43–45^. We therefore expected to suppress configural processing by turning our face-like displays upside down in Experiment 4. The chicks succeeded at discriminating between 3 and 4 upside-down face-like patterns, allowing us to exclude the possibility that failure in the previous experiment depended on the complexity of the stimuli. It is noteworthy that the difference in colour (we used orange and red coloured stimuli) cannot explain our results. The chicks, in fact, discriminated between 3 and 4 red objects in Experiment 2, but not in Experiment 1, and they discriminated between 3 and 4 orange objects in Experiment 4, but not in Experiment 3. Thus, the difference in the features and not the colour explains the chicks’ performance. The chicks were perfectly capable of processing the stimuli based on their local features when the global configuration of the face-like pattern was disrupted by inversion and no longer resembled face-like displays. Overall, our findings showed for the first time that numerical abilities can be facilitated by object identification in a non-human species. Face-like displays were used to assess number discrimination in the 3 vs 4 comparison. The system responsible for these computations can be identified as the Object File System (OFS). In fact, processing via the OFS seems to occur whenever objects disappear in spatiotemporal discontinuity (i.e. one at a time^46^). The OFS completes the initial individuation process^47^: it recognises a new object and establishes an object file in the working memory. Recognition of additional objects results in the establishment of additional object files. Individuation thus forms the basis for enumeration. Two assumptions are indispensable: the availability of free storage capacity in the working memory to collect a new object file and the fact that two objects are considered separate identities. Infants^13^ and young animals^29,48^ account for a variety of information, such as spatial–temporal cues, colour, size, shape and individual features, for object identification. Our findings support the idea that object individuation is also facilitated by the presence of individual features. In our first experiment, the differential quantitative (i.e. the overall area or perimeter) and spatial–temporal (i.e. the objects presented one at a time) cues did not facilitate 3 vs 4 number discrimination. The presence of heterogeneous stimuli constituted the crucial cue (for the benefits of congruent and redundant information on numerical cognition, see also^49,50^). In our study, facilitation occurred only if the predisposed broad preference for face-like configurations (i.e. triggering category level processing) was suppressed through display inversion. Young chicks are capable of categorising objects (e.g. friend vs foe, animate vs inanimate)^51–53^ as well as individually discriminating between them (e.g. familiar vs unfamiliar)^54^. Both processes were reported for ecological stimuli, whether natural (e.g. real conspecifics) or artificial (e.g. imprinting objects). It remains unclear whether individual features enhance object individuation in chicks under specific circumstances, even when such features are embedded in face-like configurations. Because young chicks can discriminate between real conspecifics based on the configuration of the features of their heads, the use of naturalistic stimuli may facilitate an effective integration of local and global information and could effectively facilitate non-symbolic counting. Young chicks respond to subtle differences in stimuli features and are predisposed towards realistic objects even when those objects are not naturalistic^55^. The present study employed this precocious, sophisticated model organism to investigate non-symbolic calculation. We speculate that the presence of features may cause a variation in objects’ attractiveness, affecting their representation through the OFS. Whenever processing is based on the OFS, day-old domestic chicks demonstrate the capability to discriminate among up to 3 objects^21,28^, but the numerical magnitudes that chicks can process increases whenever processing occurs through the Analogue Magnitude System (AMS)^56,57^. This numerical estimation reflects the various mechanisms underpinning numerical evaluation. The AMS computes numerousness in a collection of objects^19,46^, and its precision can be affected by the characteristics of the whole collection, such as occupancy or density^58^. The OFS is primarily dedicated to object processing, but it can also estimate numerousness precisely. Objects that are more salient would be processed more in depth, enhancing numerical estimation. The action of the OFS has been compared to that of a magnifying glass on the number line, which focuses attention on the very first, smaller, numbers and facilitating comprehension of their exact value. In contrast, the AMS permits an overall view of numerical magnitudes on the whole non-symbolic number line^19^.

Non-symbolic calculation is widespread among animals; in humans, it is considered the root of symbolic counting^59–61^. The availability of strategies for enhancing non-symbolic counting may inspire proactive interventions in loss-making mathematic cases in humans. Studies investigating the interaction between non-symbolic and symbolic number systems in humans are sparse but intriguing, as they support the idea that training non-symbolic numerical systems can improve symbolic calculations^62–66^. Such studies pave the way to interventions aimed at targeting the non-symbolic system to improve symbolic mathematical abilities^67,68^. One of the most captivating challenges of the near feature is therefore identifying ways of facilitating the acquisition of symbolic mathematics through non-symbolic numerical exercises. However, it remains unclear how and when symbolic mathematical comprehension can be facilitated using interventions on non-symbolic numerical comprehension^69^. Similarly, it also remains unclear how humans can improve their numerical reasoning using a purely non-symbolic format^21^. Clarifying those issues, besides deepening researchers’ understanding of the mechanisms involved, could provide novel insights into educational strategies for mathematical comprehension in children.

## Methods

The experiments complied with all applicable national and European laws concerning the use of animals in research and were approved by the Italian Ministry of Health (permit number 32662 of 10/01/2012). All procedures employed were examined and approved by the Ethical Committee of the University of Padua (Comitato Etico di Ateneo per la Sperimentazione Animale, C.E.A.S.A.) as well as by the Italian National Institute of Health (N.I.H).

### Subjects and rearing stimuli and rearing conditions

Sample size was determined using the pwr package in R^70^ on the basis of a previous study^21^ that employed a similar experimental procedure.

Subjects were 60 female domestic chicks (*Gallus gallus,* broiler Ross 308), which were purchased from local commercial hatcheries each week (La Pellegrina, S. Pietro in Gu, Padova, Italy). Separate groups of (N = 15) chicks were used in the four experiments. The chicks were either hatched in our laboratory or arrived at the laboratory when they were only a few hours old. Upon arrival or a few hours after hatching, they were housed in standard metal home cages (28 cm wide × 32 cm long × 40 cm high), with floors lined uniformly with white paper sheets. The temperature and humidity of the rearing room were maintained at 28–31 °C and 68%, respectively. Food and water were available ad libitum in transparent glass jars (5 cm in diameter × 5 cm high). Cages were lit using fluorescent lamps (36 W), located 45 cm above each cage. Each chick was housed and reared in a separate cage, together with seven bi-dimensional square-shaped (2.5 × 2.5 cm, less than 1 mm thick) objects made of laminated red (in Experiment 1 and 2) or orange (in Experiment 3 and 4) cardboard. We selected these colours because chicks prefer artificial social objects of red, orange or yellow hues^71,72^. These hues were employed successfully in previous studies^29,73,74^ to investigate a variety of cognitive abilities in this species. The objects were suspended by fine thread at various heights about 3–4 cm from the floor and at about 2 cm from each another. Overall, the set composed of 7 squares occupied an area of about 10 × 10 cm in the centre of each rearing cage. Objects were positioned approximately at, or slightly above, the newly hatched chicks’ head height, an optimal position for their visibility and for triggering tactile interactions from the chick (the squares could oscillate upon contact). We used a specific set of objects for each experiment. The chicks experienced the same stimuli during rearing, training and testing. In Experiment 1, the objects were all identical red squares, (Fig. 1a). In Experiment 2, the stimuli differed in terms of the relative orientation of two black segments (each 2 cm long × 0.90 mm wide) printed on both sides of each red square (Fig. 1b). We used these stimuli in a previous study^29^, which demonstrated that those features (i.e. two black segments) support individual object recognition in day-old chicks^30^. In Experiment 3, the stimuli were orange squares with three inner features triangularly arranged to produce a face-like configuration on both sides (Fig. 1c). The objects differed from each other in terms of the shape of the three features and the distances between features (eye-to-eye and eyes-to-mouth). The arrangement of the inner features (including their relative distance) is relevant for individual face perception and discrimination in human adults. Newborns can distinguish single elements of a stimulus^75^ and discriminate face-like stimuli by relying on the shapes of their internal features^76,77^. Although infants cannot use the configural information produced by the inter-feature spacing to discriminate between individual faces, naïve infant monkeys can use such information to discriminate between photographs of familiar and unfamiliar human faces^78^. For this reason, and to maximise individual differences, we designed stimuli that also differed in terms of the spacing among their inner features.

In Experiment 4, we used the same stimuli as in Experiment 3 but presented them upside-down, to disrupt configural processing. This enabled us to disentangle between the chicks’ use of local and global cues^11^.

The chicks were maintained in the rearing conditions described above from the morning (11 a.m.) of the Day 1 (Monday) to the morning (11 a.m.) of Day 4 (Thursday), when they underwent training and, about 1 h later, testing. Previous studies have shown that 3 days of exposure to such objects produces effective social attachment in this strain of chicks, so these stimuli can be considered “artificial social companions”^18^.

### Test stimuli and apparatus

At testing, stimuli identical to those used for imprinting were employed for each chick.

The experimental apparatus consisted of a circular arena (95 cm diameter, 30 cm outer wall height) with a floor uniformly lined with white plastic sheets. Within the arena, adjacent to the outer wall, was a holding box (10 × 20 × 20 cm), where the chick was confined shortly before the beginning of each trial. The box was made of opaque plastic sheets, with an open top allowing for the insertion of the chick. The side of the holding box, which faced the centre of the arena, consisted of a removable clear glass partition (20 × 10 cm). This allowed the chick, while confined, to see the inside of the arena, where one (during training) or two (during testing) blue opaque panels (16 × 8 cm) were positioned. During training, a single panel was present in the centre of the arena, in front of the holding box and 35 cm away from it. During testing, two identical panels were placed in front of the holding box, 25 cm away from it and 20 cm apart from one another.

Training and testing took place in the experimental room, where temperature and humidity were maintained at 25 °C and 70%, respectively. The room was kept dark, except for the light coming from four 40-W neon lamps placed about 80 cm above the centre of the arena. During training and testing, the experimental setting was identical for all experiments.

### Experimental procedure

#### Training

Each chick, together with a single object (randomly selected on each trial from the 7 familiar ones) was placed within the testing arena, where a single panel was positioned. The object was held at the chick’s sight height using a fine thread and kept in the space between the holding box and the panel. The chick was allowed to move around and become acquainted with the environment for about 5 min. Thereafter, the experimenter moved the object and waited for the chick to approach it before starting a new trial. At each trial, the experimenter moved the object closer to the panel until the object moved was completely hidden by the panel. This procedure was repeated a few times until the chick promptly re-joined its artificial social companion behind the panel. Thereafter, the chick was confined within the holding box, behind the transparent partition. From within the holding box, the chick could see the square being moved behind the panel. As soon as it disappeared completely, the transparent partition was lifted, and the chick was set free in the arena. Every time the chick rejoined the object, it was allowed to spend a few seconds together with it as a reward. The procedure was repeated until the chick reacted to the disappearance of the object by promptly searching for it and rejoining it behind the panel three consecutive times. All the objects presented in the rearing cage were used during each chick’s training. The presentation order was randomly determined for each subject.

Training was identical for all experiments. The only difference among them was the stimuli used.

#### Testing

Each subject underwent 20 valid trials. At the beginning of each trial, the chick was confined inside the holding box behind the transparent partition and could see the centre of the arena, where two identical screens were located. Each object from the chick’s rearing set of 7 squares was presented individually in front of the holding box, then slowly made to disappear behind either panel. The movement lasted about 3 s, and the entire procedure lasted 20 s per trial on average. About 2 s after the disappearance of one object, a second object was presented and hidden behind the panel using the same procedure. In this way, the first set of either 3 or 4 objects was hidden behind the same panel. The procedure was repeated for the second set (either 4 or 3 objects), which was hidden behind the other panel. The order of the two sets’ presentation, as well as the position of disappearance (left or right panel) was counterbalanced for each subject within each testing trial^18^. For each trial, objects were randomly assigned to either group and to the serial position in which they were presented. Thus, if any bias was present for following a specific object, this would result in an overall random performance for that chick. At testing, the chicks saw only one element at a time; the whole set of seven rearing objects was never visible at one time, nor was any subset of such objects. About 5 s after the disappearance of the final object of the second set, the transparent partition was lifted, and the chick was released into the arena.

Once free to move within the arena, the chick began to search for its rearing objects and had to choose one of the two panels to rejoin them. A trial was considered over whenever at least the head and ¾ of the chick’s body entered the area behind either panel (i.e. beyond the side edges). Only the first panel circumnavigated was scored. At the end of each trial, independent of the panel selected, the chick was allowed to spend few seconds with the subset of objects present behind the chosen panel. Thereafter the chick was positioned back within the holding box and, after 10 s, the next trial was started. If the chick did not circumnavigate either panel within 3 min, the trial was considered null, discarded and repeated immediately afterwards. Whenever a chick performed three consecutive null trials, it was placed back in its rearing cage in the presence of all its imprinting objects for at least 1 h before resuming the remaining testing trials. Chicks which scored three consecutive null trials twice during the test were excluded from the experiment. This happened for 5 chicks during the whole study (1 chick in Exp. 1, 3 in Exp. 2, 1 in Exp. 3 and 0 in Exp. 4).

During the test, the chicks’ behaviour was video-recorded. It was observed and scored using a monitor connected to a video camera located within the testing room, but away from the apparatus rather than by direct observation.

## Data Availability

The data sets are available from the corresponding author upon reasonable request.
